# Comprehensive analysis of the differential expression and prognostic value of COL1A2 in colon adenocarcinoma

**DOI:** 10.18632/aging.204261

**Published:** 2022-09-02

**Authors:** Jian-Jiang Jin, Ting Zheng, Xiao-Xia Xu, Lei Zheng, Fang-Yuan Li, Xing-Xing Li, Li Zhou

**Affiliations:** 1 Department of Medical Oncology, The First People's Hospital of Linping, Hangzhou 311103, Zhejiang, China

**Keywords:** COL1A2, colon adenocarcinoma, biomarker, prognosis

## Abstract

Background: Colon adenocarcinoma (COAD) is a highly heterogeneous disease, which is the second most common cancer in females and third in males. Collagen type I alpha 2 (COL1A2) has been documented to be involved in the carcinogenesis of multiple tumors; however, the expression and prognostic significance of COL1A2 and its underlying mechanism in COAD remains unclarified.

Materials and Methods: The general profile of COL1A2, its expression pattern, and prognostic value were systematically assessed through various bioinformatics tools. The protein level of COL1A2 was verified in COAD patients using immunohistochemistry analysis. In addition, enrichment analyses were performed to explore the possible regulatory pathways of COL1A2 in COAD.

Results: The mRNA and protein levels of COL1A2 were significantly increased in COAD than that in normal tissues (*P* < 0.05). The COL1A2 expression tended to increase along with cancer stages and nodal metastasis status in COAD, while the promoter methylation levels of COL1A2 might negatively related to its mRNA expression. Survival analysis showed that COL1A2 was a reliable predictor for distinguishing the status of disease-specific survival (DSS), overall survival (OS), and progression-free survival (PFS), and might serve as a robust independent prognostic biomarker for DSS and OS in COAD patients (*P* < 0.05). The enrichment analysis showed focal adhesion as the most possible regulatory pathway by COL1A2.

Conclusion: Collectively, COL1A2 functioned as an independent prognostic biomarker and might be a potential therapeutic target in COAD.

## INTRODUCTION

Colon adenocarcinoma (COAD) is the third most frequent cancer and the second main trigger of cancer-related death worldwide [1]. COAD is a heterogeneous digestive tract tumor characterized by dysregulated differentiation, proliferation, and apoptosis of intestinal epithelial cells [2]. According to statistics, 1.93 million new cases of COAD were identified, and 935,173 people died of this kind of cancer in 2020 [3]. It is the second most common cancer in females and third in males, which caused more than 500,000 mortalities globally each year [4]. Most COAD cases are attributable to environmental factors such as dietary and chemicals, leading to genetic instabilities in intestinal cells, specific intestinal symbionts, and pathogens [5]. Over 50% of COAD-related deaths are closely associated with risk factors including alcohol consumption, smoking, and lack of exercise [6]. Currently, surgery, radiotherapy, chemotherapy, targeted therapy, or their combination are available therapeutic options administrated to COAD patients [7]. In spite of the continuous innovations and significant advances in treatment techniques, the 5-year overall survival (OS) rate remains unsatisfactory at only 50%–65% and it is still incurable for advanced or metastatic COAD patients [8]. Therefore, it is imperative to understand the underlying mechanism responsible for COAD and develop a potential biomarker to prolong the survival time of COAD patients.

Collagen is the primary component of the extracellular matrix (ECM) and the most abundant type is collagen type I [9]. Collagen type I as a structural protein is observed in connective tissues such as tendon, bone, and skin [10]. It is a triple helix composed of two chains of collagen type I alpha 1 (COL1A1) and one chain of collagen type I alpha 2 (COL1A2) [10]. The structural integrity and coordinated biosynthesis of these chains are critical for tissue morphogenesis, growth, homeostasis, and repair [11]. Changes in collagen type I synthesis occur in embryogenesis, wound healing, and some pathological conditions, including fibrosis of the kidney, lung, and liver, scleroderma as well as cancers [12]. Moreover, the expression level of the fibrosis-related protein COL1A2 was obviously upregulated in the colonic tissue with intestinal fibrosis [13, 14]. Previous studies have demonstrated the involvement of COL1A2 in the cancer process, which can be both stimulatory and inhibitory. COL1A2 accelerated the development and angiogenesis of melanoma and medulloblastoma [15, 16]. On the other hand, COL1A2 suppressed the proliferation, migration, and invasion of bladder cancer cells [17]. Nevertheless, the prognostic role of COL1A2 and the actual significance across the various clinicopathological parameters such as age, gender, weight, histological subtypes, cancer stage, and nodal metastasis status in COAD have not been systemically studied yet.

Herein, multiple bioinformatics tools were adopted and the dataset of well-established cancer data from various demographic and clinicopathological patients was downloaded for comprehensive research of COAD. Firstly, we analyzed the expression and prognostic values of COL1A2 in COAD. Further, the underlying mechanisms of COAD based on COL1A2-related genes were explored by enrichment analysis.

## MATERIALS AND METHODS

### The general profile of COL1A2

The normalized RNA sequencing data based on TCGA Pan-Cancer were downloaded from the UCSC Xena database (https://xenabrowser.net/datapages/) to analyze the difference in COL1A2 mRNA expression levels between various tumors and normal tissues. The data were used to extract the mRNA expression values of COL1A2 and the tumors with less than three samples were deleted. Differential COL1A2 mRNA expression analyses in each tumor type were performed using the Student’s *t*-test. *P* < 0.05 was considered to be statistically significant. Then, the Human Protein Atlas (HPA) (https://www.proteinatlas.org/) database was utilized to examine the localization of COL1A2 protein in human tumor cells in the “SUBCELL” column. The HPA database aims to map all the human proteins in cells, tissues, and organs using an integration of various omics technologies. As the world’s largest and most comprehensive resource for exploring the impact of somatic mutations in human cancer, the Catalogue of Somatic Mutations in Cancer (COSMIC) (https://cancer.sanger.ac.uk/cosmic) was applied for analyzing the different mutation types related to the COL1A2 gene.

### Expression analysis of COL1A2 in COAD

The expression data and corresponding clinical information in TCGA-COAD were obtained from the UCSC Xena database. The differential gene expression levels of COL1A2 in COAD and normal tissues were compared using unpaired and paired *t*-tests. A *P*-value less than 0.05 was regarded as the threshold of significance. Through the HPA database, we evaluated the protein levels of COL1A2 in COAD and normal tissues by immunohistochemical analysis, which was validated in an additional population with COAD.

Subsequently, the expression and promoter methylation level of the COL1A2 gene in COAD based on clinicopathological parameters such as age, gender, weight, histological subtypes, cancer stage, and nodal metastasis status was assessed using the UALCAN web server (http://ualcan.path.uab.edu/analysis.html), which is an interactive database for analyzing cancer OMICS data.

### Prognosis analysis of COL1A2 in COAD

The relationship between COL1A2 expression and disease-free interval (DFI), disease-specific survival (DSS), overall survival (OS), and progression-free survival (PFS) in COAD was examined in the Gene Set Cancer Analysis (GSCA) (http://bioinfo.life.hust.edu.cn/GSCA/#/) which is an integrated database for genomic and immunogenomic gene set cancer analysis. *P* < 0.05 indicates statistical significance. Besides, the value of COL1A2 in distinguishing the survival status with regard to DFI, DSS, OS, and PFS was determined by the receiver operating characteristic (ROC) curve based on the TCGA-COAD data. The computed area under the curve (AUC) value ranging from 0.5 to 1.0 indicated the discrimination ability from 50–100%. Moreover, Cox regression analysis was performed to evaluate the independent prognostic value of COL1A2 by SPSS software (version 23.0), and *P* < 0.05 was considered to be statistically significant. Further, the R package “rms” was adopted to construct the nomogram and plot the calibration curves to predict 1-, 3-, and 5- year OS and DSS for COAD patients. The concordance index (C-index) was used to evaluate the predictive accuracy of the nomogram. C-index from 0.50 to 0.70 (lower accuracy), 0.71 to 0.90 (medium accuracy), above 0.90 (high accuracy) [18].

### Enrichment analysis

To explore the underlying mechanism of COL1A2 in COAD, the TCGA-COAD samples were divided into high-COL1A2 and low-COL1A2 expression groups based on the median value of the COL1A2 gene. The DEGs between two expression groups were screened using the “limma” package. |Log2 Fold change (FC)| Cutoff >1 and *P* < 0.05 were set as the criterion of significant differences.

Following this, we conducted Gene Ontology (GO) annotations and the Kyoto Encyclopedia of Genes and Genomes (KEGG) pathway of the significant DEGs using the R package “cluster profiler”. A *P*-value less than 0.05 was considered statistically significant. Gene Set Enrichment Analysis (GSEA) was conducted to further investigate the signaling pathways regulated by COL1A2-related genes in COAD. The gene set permutations were performed 1000 times. Clusters with a false discovery rate (FDR) *q*-value < 0.05 and *P*-value < 0.05 were identified as significant.

Subsequently, we defined the enrichment level of a pathway in COAD samples as the single-sample gene-set enrichment analysis (ssGSEA) score [19]. The gene set represents the collection of all marker genes of a pathway. The association of the COL1A2 mRNA expression with the enrichment levels (ssGSEA scores) of the pathway was evaluated using Pearson’s correlation test.

### Data sharing statement

The datasets used and/or analyzed during the current study are available from the corresponding authors upon reasonable request.

## RESULTS

### Patient characteristics

The TCGA-COAD dataset contains 293 samples with complete survival and gene expression data. As shown in Table 1, there were 46 patients (15.7%) diagnosed at the age of fewer than 50 years and 247 patients (84.3) over 50 years. Totally 75 patients (33.9%) had body mass index (BMI) < 25, and 133 patients (45.4%) were females. N0, N1, and N2 were found in 174 (59.4%), 71 (24.2%), and 48 (16.4%) patients, separately. Patients at stages I, II, III, and IV were 48 (17.0%), 114 (40.3%), 82 (29.0%), and 39 (13.7%), respectively. Besides, 250 patients were diagnosed with adenocarcinoma (85.9%). The TCGA-COAD dataset includes 42 paired samples.

**Table 1:** The clinicopathological characteristics of patients in colon adenocarcinoma.

**Clinical characteristics**	**Total number (%)**
Age	
≤50	46 (15.7)
>50	247 (84.3)
Body mass index	
<25	75 (33.9)
≥25	146 (66.1)
Gender	
Female	133 (45.4)
Male	160 (54.6)
Nodal metastasis status	
N0	174 (59.4)
N1	71 (24.2)
N2	48 (16.4)
Cancer stage	
Stage I	48 (17.0)
Stage II	114 (40.3)
Stage III	82 (29.0)
Stage IV	39 (13.7)
Subtype	
Adenocarcinoma	250 (85.9)
Mucinous adenocarcinoma	41 (14.1)

### The general profile of COL1A2

**Figure 1:**
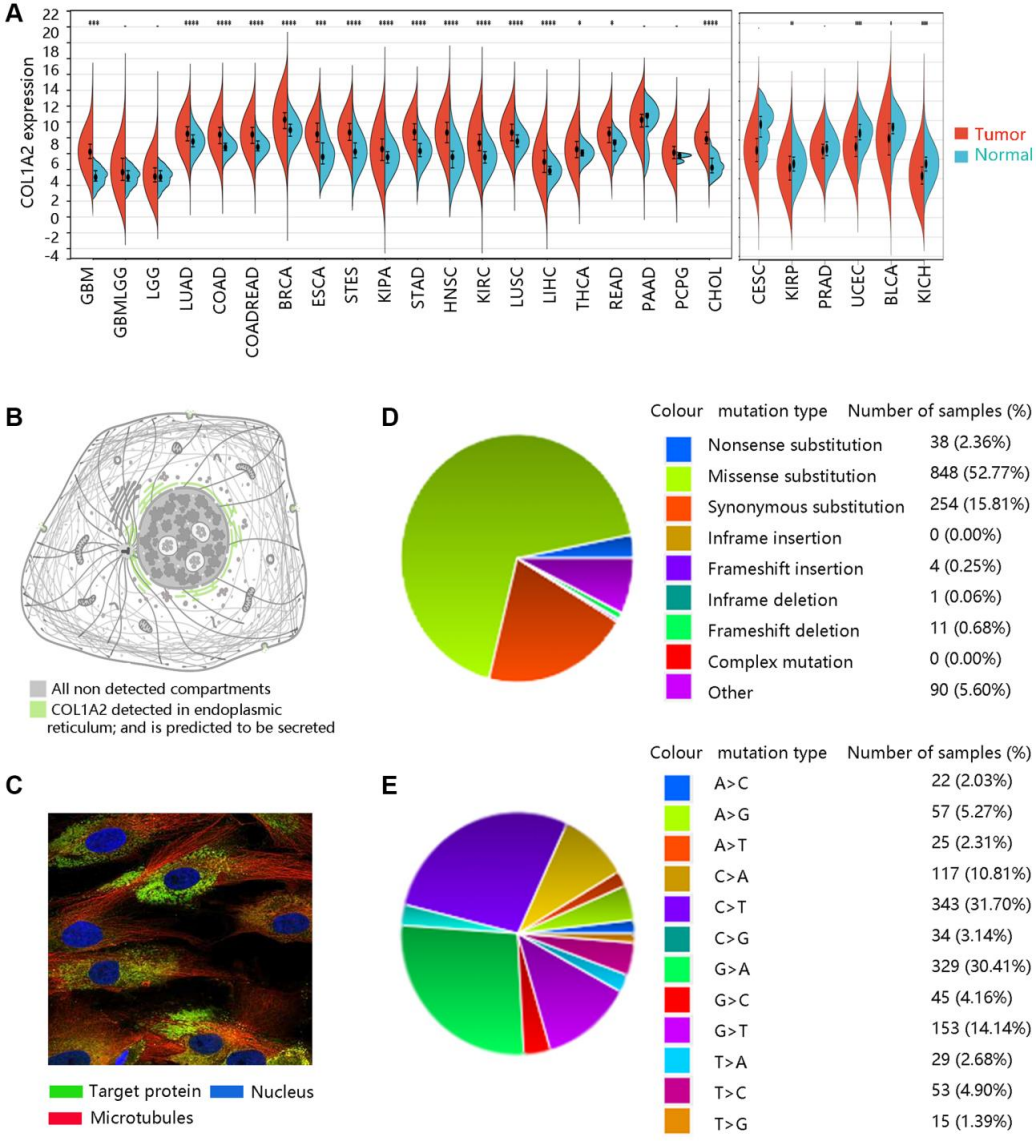
**The basic characteristics of COL1A2.** (**A**) The COL1A2 gene expression among multiple cancers. ^*^P < 0.05, ^**^P < 0.01, ^***^P < 0.001. (**B**) Subcellular location and (**C**) immunofluorescence image of COL1A2 protein in human tumor cells. (**D**) Summary of different types of mutations related to the COL1A2 gene. (**E**) Various types of substitutional mutations.

We firstly analyzed the expression levels of COL1A2 among various cancer types (Figure 1A). Subcellular location and immunofluorescence image of COL1A2 expression in human tumor cells were retrieved from the HPA (Figure 1B, 1C). The COL1A2 gene mutation in pan-cancer was evaluated through the COSMIC database. A total of 1246 samples were recorded for mutations, among which the missense substitution (52.77%) had the highest proportion, followed by synonymous substitution (15.81%), and other types (8.95%) (Figure 1D). Figure 1E presented the breakdown of different substitution mutations, exhibiting the highest type of C > T (31.70%), and the lowest type of T > G (1.39%). Of note, no mutation of the COL1A2 gene occurred in COAD.

### Expression analysis of COL1A2

**Figure 2:**
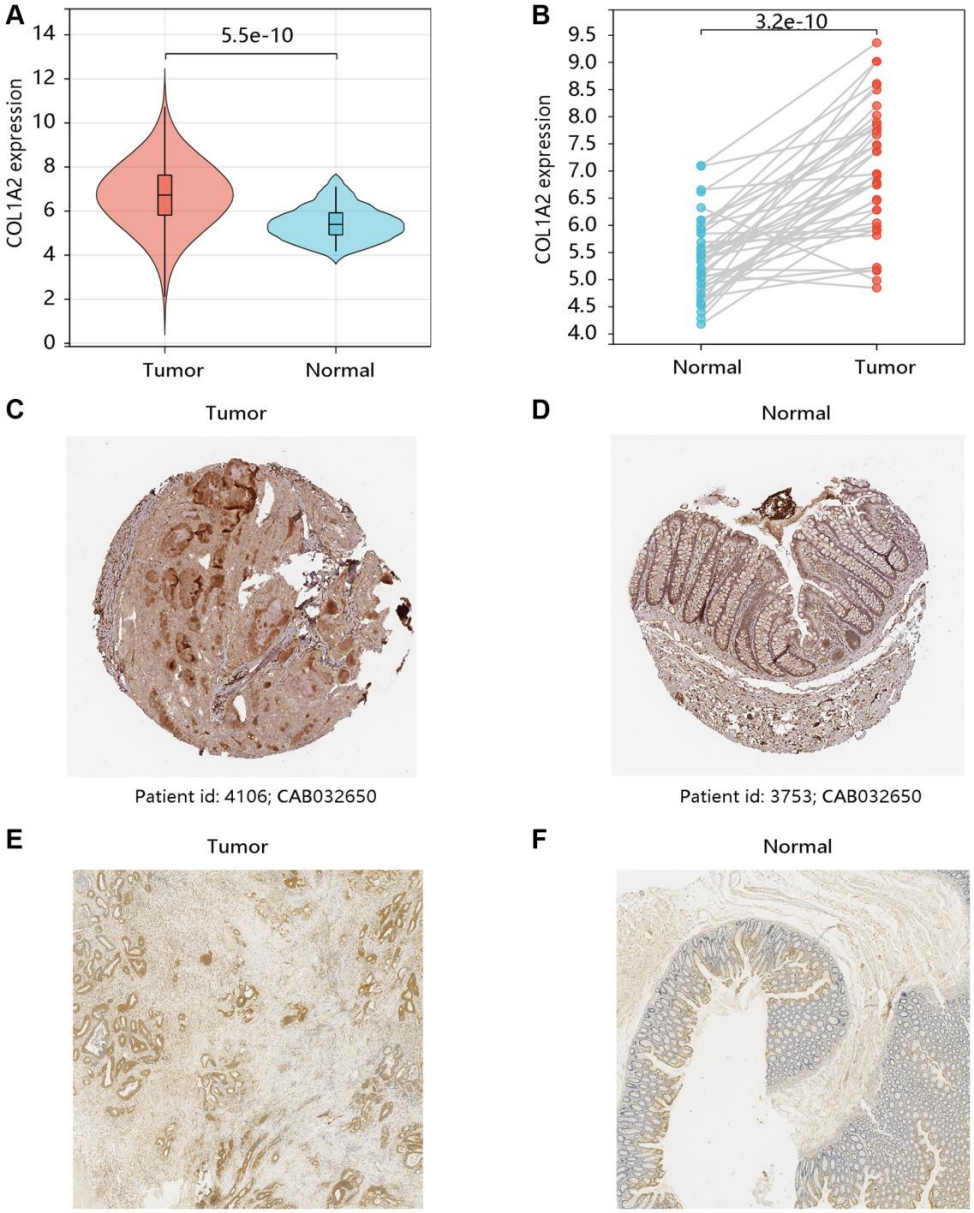
**The mRNA and protein levels of COL1A2 in colon adenocarcinoma (COAD).** The differential gene expression of COL1A2 in COAD and normal tissues using (**A**) unpaired and (**B**) paired *t*-tests. The COL1A2 protein levels in (**C**) COAD and (**D**) normal colon tissues using the HPA database. The COL1A2 proteins in (**E**) COAD and (**F**) normal colon tissues by the immunohistochemistry analysis.

Based on the TCGA-COAD data, the mRNA expression levels of COL1A2 in COAD and normal tissues were compared. Both unpaired and paired *t*-tests demonstrated that COL1A2 gene expression was remarkably upregulated in the COAD compared with that in normal tissues (all *P* < 0.001) (Figure 2A, 2B). The HPA database showed the different COL1A2 protein expressions in COAD and normal colon samples (Figure 2C, 2D). The immunohistochemistry analysis validated the higher protein level of COL1A2 in COAD tissue (Figure 2E, 2F).

### Transcriptional expression and epigenetic regulation of COL1A2 across different clinicopathological factors

**Figure 3:**
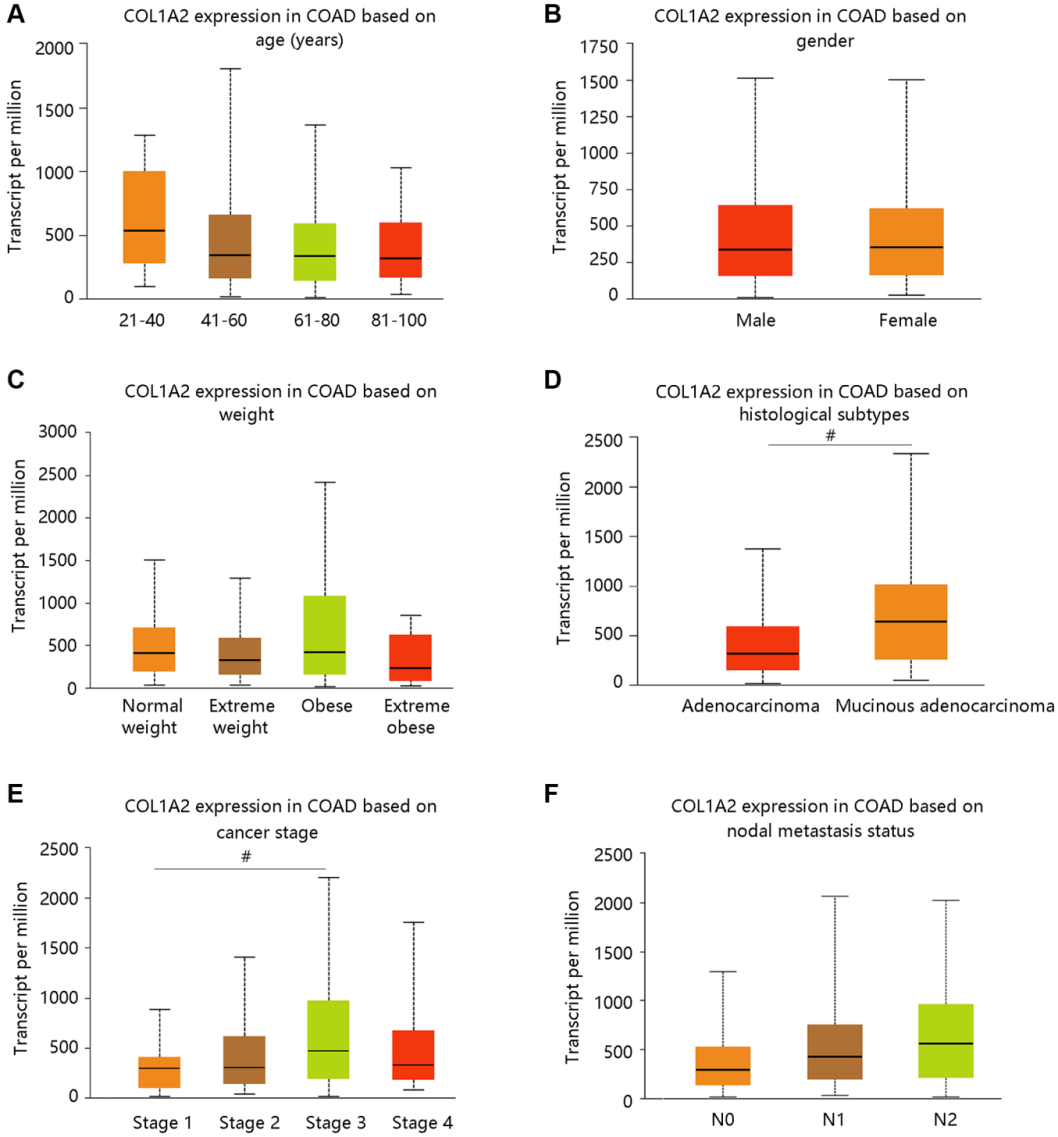
**The COL1A2 expression in colon adenocarcinoma based on various clinicopathological parameters.** (**A**) Age (years). (**B**) Gender. (**C**) Weight. Normal weight: 18.5 ≤BMI < 25; Overweight: 25 ≤ BMI < 30; Obese: 30 ≤ BMI < 40; Extreme obese: BMI > 40. Abbreviation: BMI: body-mass-index. (**D**) Histological subtypes. (**E**) Cancer stage. (**F**) Nodal metastasis status. ^#^*P* < 0.05.

We have observed the elevated mRNA level of the COL1A2 in COAD in the earlier section, and then we evaluated the COL1A2 expression in COAD based on various clinicopathological characteristics like age, gender, weight, histological subtypes, cancer stage, and nodal metastasis status (Figure 3A–3F). Patients with mucinous adenocarcinoma had remarkably higher COL1A2 mRNA expression than those with adenocarcinoma (*P* < 0.05) (Figure 3D). It tended to increase the COL1A2 expression at advanced cancer stages (stage 1 < stage 2 < stage 3) and COL1A2 expression increased along with the nodal metastasis status (N0 < N1 < N2) (Figure 3E, 3F).

**Figure 4:**
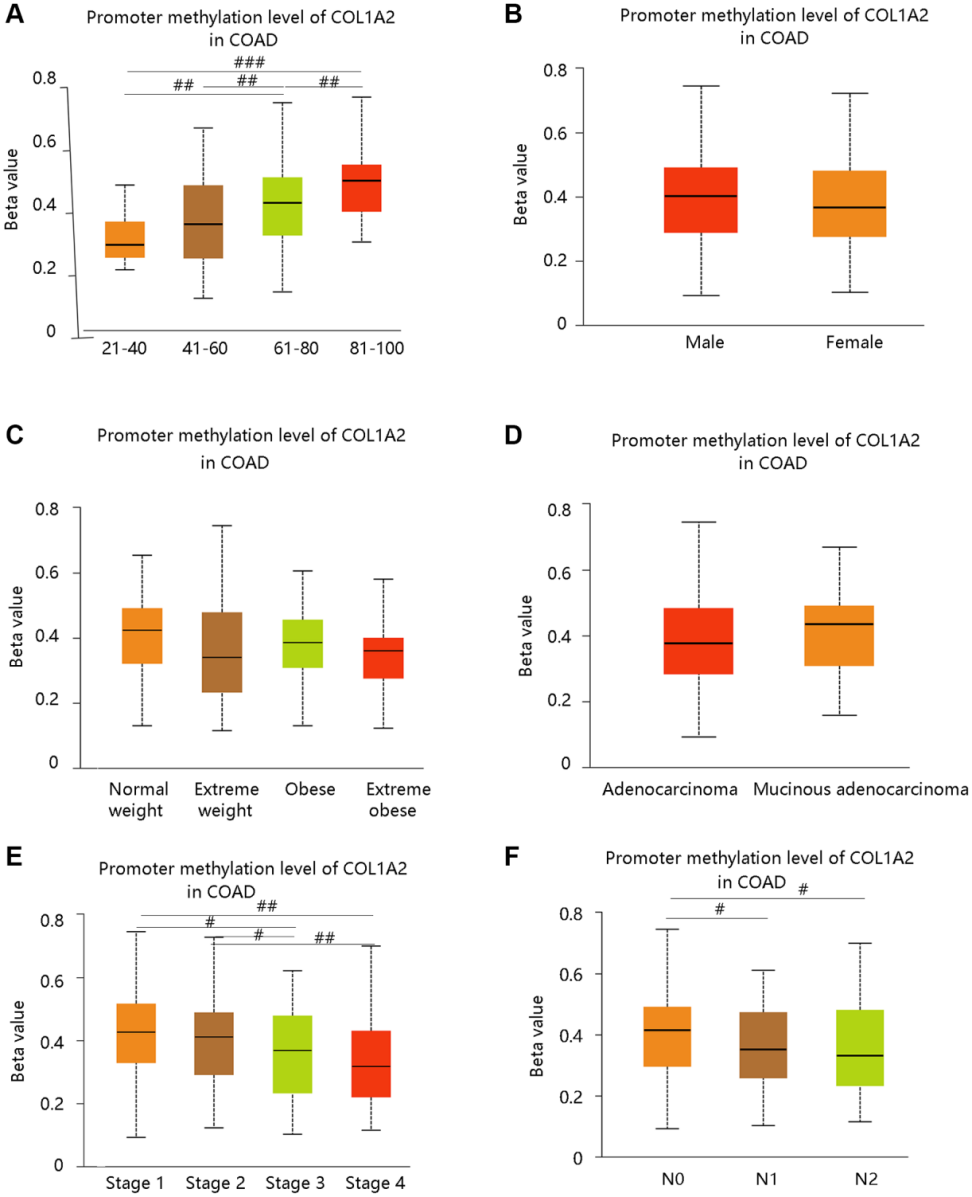
**The COL1A2 promoter methylation level in colon adenocarcinoma based on various clinicopathological parameters.** (**A**) Age. (**B**) Gender. (**C**) Weight. Normal weight: 18.5 ≤ BMI < 25; Overweight: 25 ≤ BMI < 30; Obese: 30 ≤ BMI<40; Extreme obese: BMI > 40. Abbreviation: BMI: body-mass-index. (**D**) Histological subtypes. (**E**) Cancer stage. (**F**) Nodal metastasis status. ^#^*P* < 0.05; ^##^*P* < 0.01; ^###^*P* < 0.001.

DNA methylation is closely related to the development of cancer in the human body [20]. From our data, the promoter methylation of COL1A2 was significantly related to age, cancer stage, and nodal metastasis status, but had no notable relation with gender, weight, and histological subtypes (Figure 4A–4F). Intriguingly, the promoter methylation level of COL1A2 decreased with the development of cancer stages (stage 4 < stage 3 < stage 2 < stage 1) and nodal metastasis status (N2 < N1 < N0) (Figure 4E, 4F). The above findings suggested that the promoter methylation of COL1A2 might have a negative relation with its mRNA expression in COAD.

### COL1A2 expression predicted survival status and served as an independent prognostic biomarker in COAD

**Figure 5:**
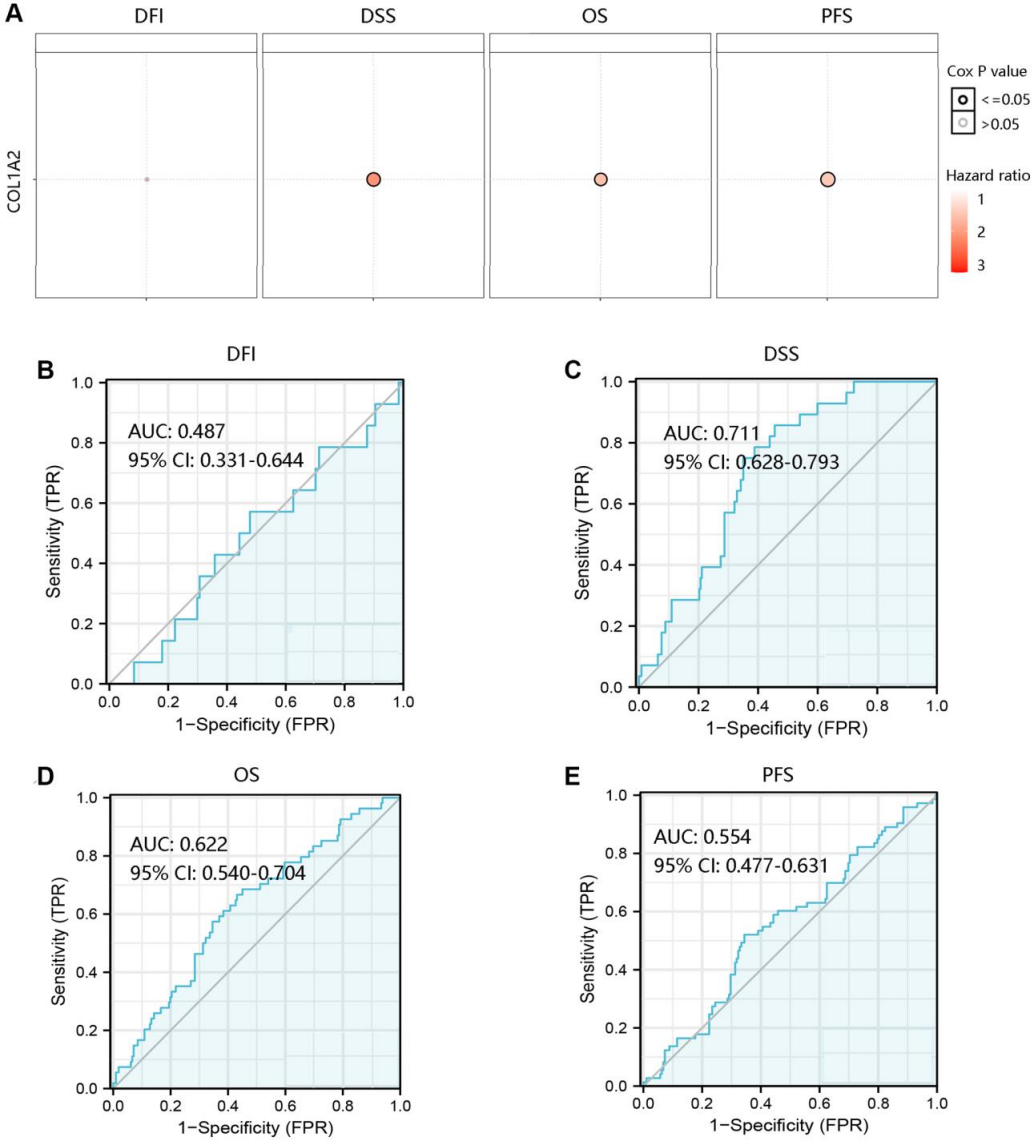
**Prognostic and predictive analysis of COL1A2 in colon adenocarcinoma (COAD).** (**A**) The effect of COL1A2 on DFI, DSS, OS, and PFS in COAD. The value of COL1A2 in predicting survival status of (**B**) DFI, (**C**) DSS, (**D**) OS, and (**E**) PFS. Abbreviations: DFI: disease-free interval; DSS: disease-specific survival; OS: overall survival; PFS: progression-free survival; AUC: area under the curve; 95% CI: 95% confidence interval; FPR: false-positive rate; TPR: true-positive rate.

To investigate the effect of COL1A2 mRNA expression on the clinical outcomes of COAD patients, the GSCA database was employed to analyze the association of COL1A2 with DFI, DSS, OS, and PFS. High COL1A2 expression led to a worse prognosis except for DFI (Figure 5A). Then, the ROC curves of COL1A2 expression were drawn to examine the value of COL1A2 in predicting the survival status. Similarly, COL1A2 could not predict DFI status (AUC = 0.487), but exhibited the satisfactory performance in predicting survival status of DSS (AUC = 0.711), OS (AUC = 0.622), and PFS (AUC = 0.554) (Figure 5B–5E).

To further probe into the independent prognostic value of COL1A2 in predicting DSS, OS, and PFS of COAD patients, Cox regression analysis was conducted using the TCGA-COAD data. The results showed that age, N1, N2, stage IV, and COL1A2 had a significant relationship with DSS in COAD patients (all *P* < 0.05), but other clinical factors were not associated with DSS in the univariate analysis. When these variables were integrated into multivariate Cox regression analysis, age and COL1A2 were independent prognostic factors (all *P* < 0.05) (Table 2). In addition, N2, stage III, stage IV, and COL1A2 were closely connected with OS (all *P* < 0.05), while only COL1A2 (*P* < 0.05) was still significantly associated with OS in the multivariate analysis (Table 3). However, COL1A2 could not independently predict the PFS of COAD patients (*P* < 0.05) (Table 4). These results indicated that high COL1A2 expression was an independent prognostic factor for predicting worse DSS and OS in COAD.

**Table 2:** Cox regression analyses of disease-specific survival in colon adenocarcinoma patients.

**Variable**	**Univariate analysis**	***P*-value**	**Multivariate analysis**	***P*-value**
	**HR (95% CI)**		**HR (95% CI)**	
Age	0.970 (0.944–0.997)	0.031	0.954 (0.912–0.999)	0.043
BMI	0.991 (0.940–1.043)	0.721	0.985 (0.894–1.086)	0.763
Gender				
Male vs. female	1.552 (0.714–3.371)	0.267	0.925 (0.292–2.927)	0.894
N status				
N1 vs. N0	3.840 (1.540–9.577)	0.004	0.333 (0.024–4.629)	0.413
N2 vs. N0	5.302 (2.040–13.780)	0.001	0.630 (0.049–8.079)	0.722
Cancer stage
II vs. I	1.399 (0.151–12.914)	0.767	9931.693 (0.000-Inf)	0.943
III vs. I	4.754 (0.593–38.099)	0.142	48372.047 (0.000-Inf)	0.934
IV vs. I	23.294 (3.003–180.674)	0.003	244290.187 (0.000-Inf)	0.924
Subtype
Mucinous adenocarcinoma vs. adenocarcinoma	0.759 (0.228–2.529)	0.653	1.006 (0.186–5.432)	0.994
COL1A2	1.954 (1.481–2.579)	<0.001	1.674 (1.040–2.696)	0.034

Abbreviations: HR: hazard ratio; 95% CI: 95% confidence interval; BMI: body mass index; N status: nodal metastasis status.

**Table 3:** Cox regression analyses of overall survival in colon adenocarcinoma patients.

**Variable**	**Univariate analysis**	***P*-value**	**Multivariate analysis**	***P*-value**
	**HR (95% CI)**		**HR (95% CI)**	
Age	1.011 (0.990–1.033)	0.296	1.008 (0.981–1.035)	0.572
BMI	0.943 (0.886–1.005)	0.070	0.933 (0.870–1.002)	0.056
Gender
Male vs. female	1.471 (0.846–2.560)	0.172	0.918 (0.439–1.922)	0.821
N status
N1 vs. N0	1.711 (0.883–3.317)	0.112	0.317 (0.033–3.078)	0.322
N2 vs. N0	3.430 (1.815–6.481)	<0.001	1.200 (0.127–11.364)	0.874
Cancer stage
II vs. I	6.078 (0.807–45.787)	0.080	13156.010 (0.000-Inf)	0.888
III vs. I	10.318 (1.372–77.596)	0.023	36880.177 (0.000-Inf)	0.876
IV vs. I	25.016 (3.248–192.678)	0.002	108029.274 (0.000-Inf)	0.863
Subtype
Mucinous adenocarcinoma vs. adenocarcinoma	1.388 (0.694–2.775)	0.354	1.927 (0.799–4.645)	0.144
COL1A2	1.585 (1.297–1.937)	<0.001	1.298 (1.006–1.674)	0.045

Abbreviations: HR: hazard ratio; 95% CI: 95% confidence interval; BMI: body mass index; N status: nodal metastasis status.

**Table 4:** Cox regression analyses of progression-free survival in colon adenocarcinoma patients.

**Variable**	**Univariate analysis**	***P*-value**	**Multivariate analysis**	***P*-value**
	**HR (95% CI)**		**HR (95% CI)**	
Age	0.985 (0.968–1.003)	0.102	0.992 (0.970–1.015)	0.506
BMI	0.991 (0.963–1.019)	0.515	0.989 (0.956–1.024)	0.547
Gender
Male vs. female	1.371 (0.854–2.203)	0.192	1.023 (0.569–1.839)	0.938
N status
N1 vs. N0	1.640 (0.933–2.881)	0.086	0.698 (0.138–3.536)	0.664
N2 vs. N0	3.859 (2.201–6.766)	<0.001	2.053 (0.412–10.224)	0.380
Cancer stage
II vs. I	2.059 (0.785–5.396)	0.142	1.788 (0.597–5.351)	0.299
III vs. I	3.072 (1.157–8.161)	0.024	1.777 (0.263–12.005)	0.555
IV vs. I	9.563 (3.498–26.141)	<0.001	6.725 (1.156–39.130)	0.034
Subtype
Mucinous adenocarcinoma vs. adenocarcinoma	0.684 (0.313–1.498)	0.342	0.941 (0.395–2.240)	0.891
COL1A2	1.307 (1.106–1.544)	0.002	1.146 (0.924–1.422)	0.215

Abbreviations: HR: hazard ratio; 95% CI: 95% confidence interval; BMI: body mass index; N status: nodal metastasis status.

**Figure 6:**
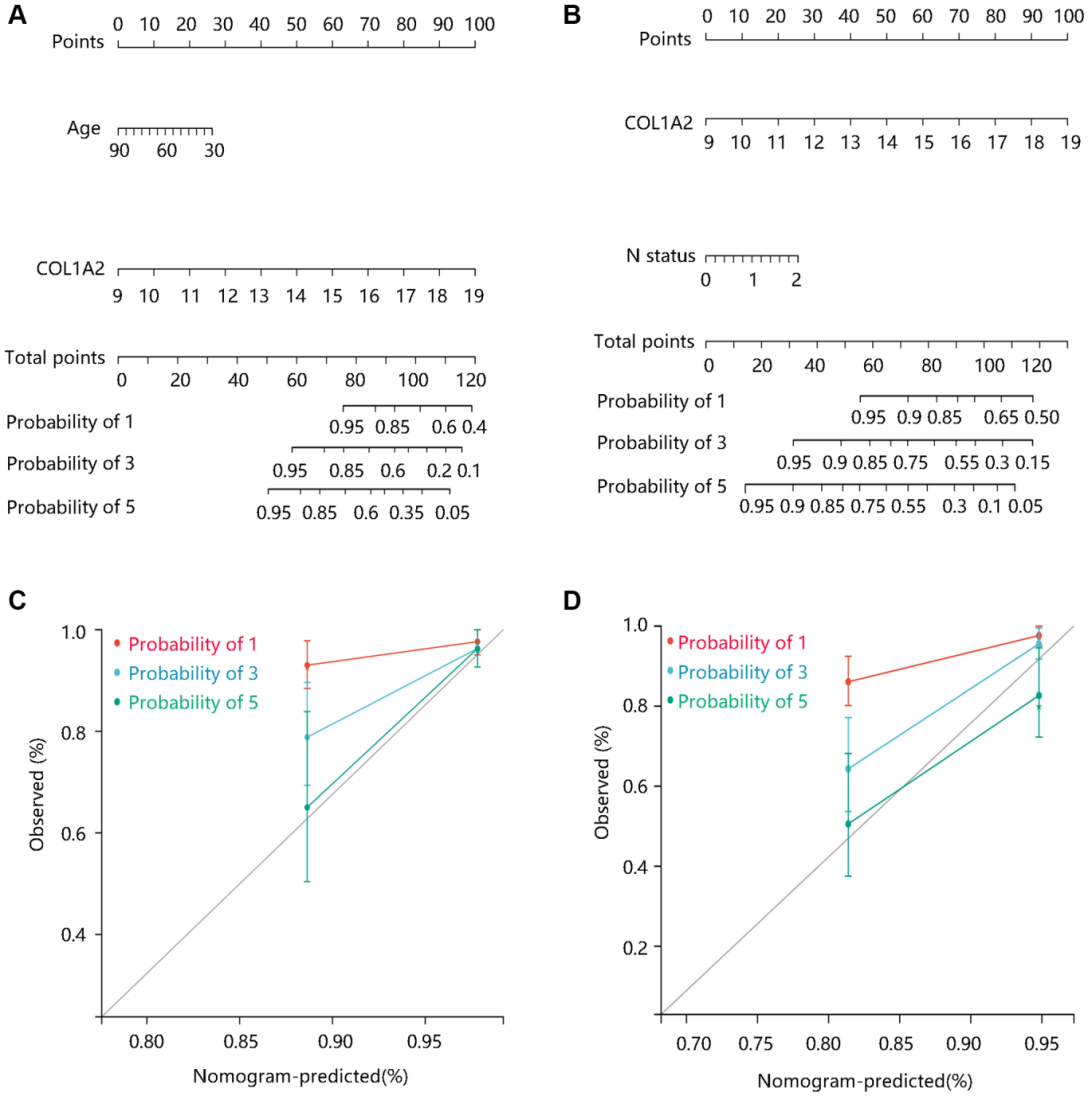
**The construction and evaluation of nomogram models.** Nomogram models based on Cox regression analysis results in terms of (**A**) DSS, and (**B**) OS. The calibration curves for the (**C**) DSS nomogram model and (**D**) OS nomogram model. Abbreviations: DSS: disease-specific survival; OS: overall survival.

To better predict the prognosis of COAD patients, two nomogram models on basis of the multivariate Cox regression analysis results were constructed and calibration curves were drawn to evaluate their efficiency. Two statistically significant prognostic factors, age, and COL1A2 were included in the nomogram model based on DSS, which had a C-index of 0.765 (Figure 6A). N status and COL1A2 were enrolled in the construction of the nomogram model based on OS, which had a C-index of 0.734 (Figure 6B). The calibration curves for 1-, 3-, and 5-year DSS and OS of COAD patients were presented in Figure 6C, and Figure 6D, respectively. Importantly, the calibration curve showed good agreement between prediction and observation in the 5-year survival probability. These findings revealed that the nomogram models had high efficiency in anticipating DSS and OS of COAD patients.

### Enrichment analysis

**Figure 7:**
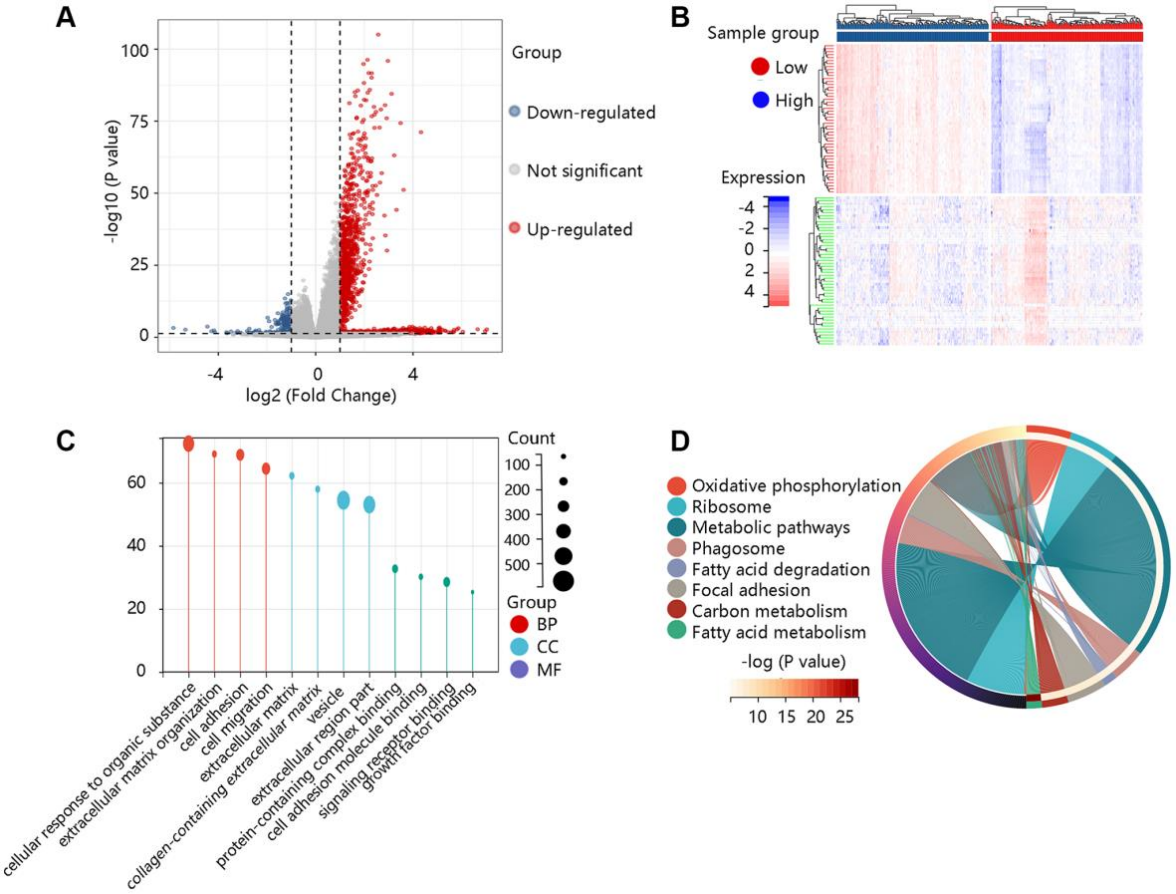
**Functional enrichment analysis of the differentially expressed genes (DEGs).** (**A**) The volcano plot exhibited the DEGs between high- and low- COL1A2 expression groups. (**B**) The heat map showed the top 50 significant DEGs. (**C**) The gene ontology annotations of the DEGs. (**D**) The KEGG pathway enrichment of the DEGs.

To elucidate the pathological function of COL1A2, the DEGs between the high- and low- COL1A2 expression groups were firstly identified as shown in the volcano plot (Figure 7A) and heat map (Figure 7B). According to the selection criterion, the significant DEGs were used for GO and KEGG enrichment analyses. GO annotations predicted the functional effect of the target genes in three aspects: biological process (BP), cellular component (CC), and molecular function (MF). We found that cellular response to an organic substance, extracellular matrix organization, extracellular matrix, vesicle, signaling receptor binding, and growth factor binding were significantly regulated in COAD (Figure 7C). The KEGG enrichment analysis showed that the pathways related to the functions of COL1A2 interactive genes were oxidative phosphorylation, phagosome, focal adhesion, fatty acid metabolism (Figure 7D).

**Figure 8:**
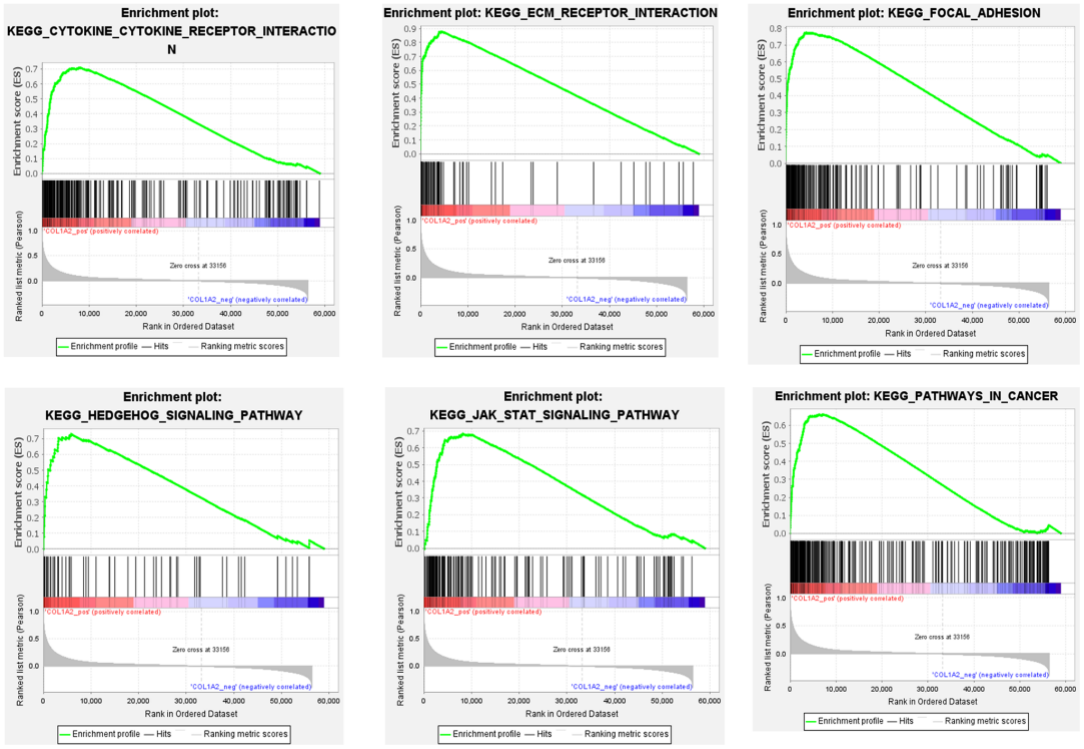
Gene set enrichment analysis of COL1A2 in colon adenocarcinoma.

To further reveal the underlying mechanism of COL1A2 in COAD, GSEA was performed on gene expression microarray data of TCGA-COAD. The results showed that the top six positive pathways were cytokine-cytokine receptor interaction (NES = 2.192, *P* < 0.001), ECM receptor interaction (NES = 2.260, *P* < 0.001), focal adhesion (NES = 2.334, *P* < 0.001), Hedgehog signaling pathway (NES = 2.237, *P* < 0.001), JAK-STAT signaling pathway (NES = 2.228, *P* < 0.001), and pathways in cancer (NES = 2.277, *P* < 0.001) (Figure 8) (Table 5). Combined with the previous enrichment analysis results, focal adhesion was the consistent pathway.

**Table 5:** Gene set enrichment analysis of COL1A2 in colon adenocarcinoma.

**KEGG name**	**ES**	**NES**	**NOM *p*-value**	**FDR *q*-value**
Cytokine-cytokine receptor interaction	0.709	2.192	<0.001	0.002
ECM receptor interaction	0.877	2.260	<0.001	0.002
Focal adhesion	0.773	2.334	<0.001	0.001
Hedgehog signaling pathway	0.729	2.237	<0.001	0.002
JAK-STAT signaling pathway	0.684	2.228	<0.001	0.002
Pathways in cancer	0.660	2.277	<0.001	0.002

Abbreviations: ES: enrichment score; NES: normalized enrichment score; NOM: nominal; FDR: false discovery rate.

**Figure 9:**
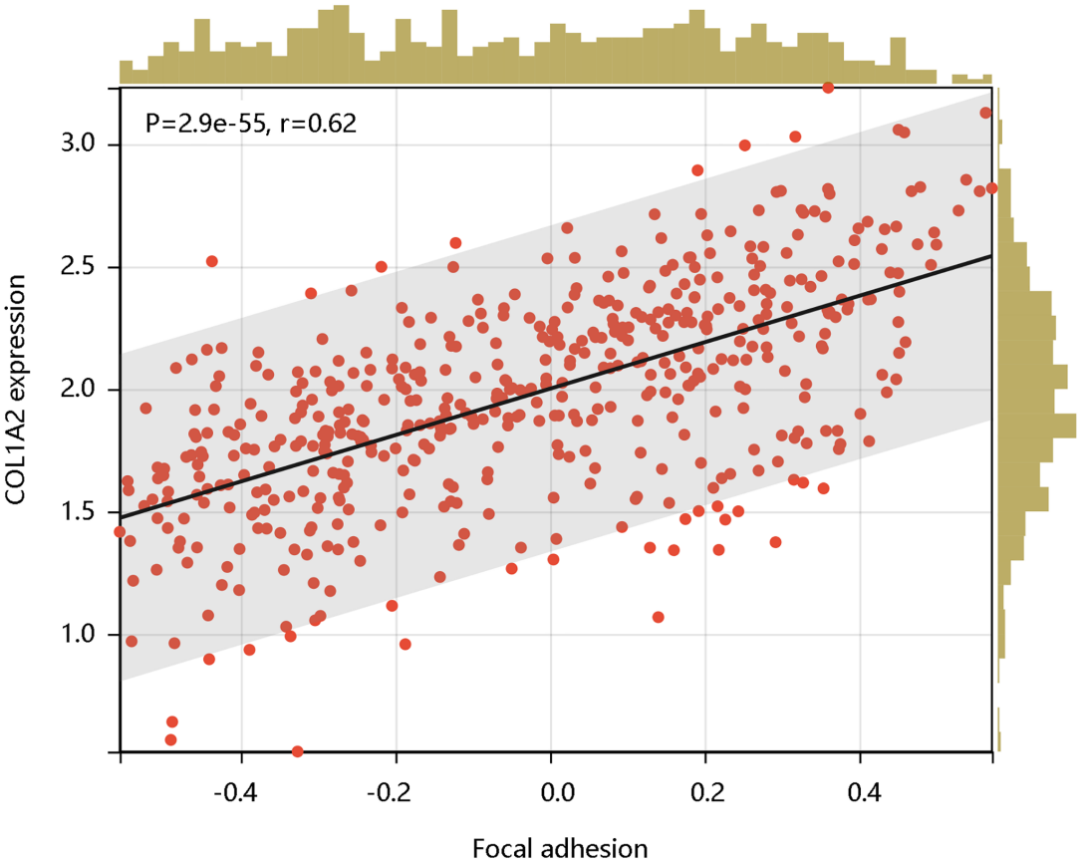
The significant positive correlation of COL1A2 mRNA expression with the enrichment levels of the focal adhesion pathway.

Following this, we analyzed the enrichment levels of the focal adhesion pathway using ssGSEA. The COL1A2 gene expression level had a significant positive correlation with the enrichment levels (ssGSEA scores) of the focal adhesion pathway (*P* < 0.001) (Figure 9). Therefore, the COL1A2 might affect the development of COAD via positive regulation of the focal adhesion pathway.

## DISCUSSION

COAD is a heterogeneous disease always occurring in the elderly [21]. Besides, COAD is highly invasive and is easily spread to other organs with the liver as the most common site of metastasis. Although significant achievements have been made in the treatment strategies, the 5-year survival rate is still low and the 5-year OS for metastatic patients is approximately 13% [22]. Hence, searching for novel molecular biomarkers for carcinogenesis and pathogenesis of COAD was essential for the COAD diagnosis and treatment. COL1A2 encodes the alpha 2 chain of collagen type I which is the main ECM component of bone and skin [23]. It has been demonstrated that the abnormal COL1A2 mRNA expression led to a worse prognosis of gastric cancer patients and enhances the proliferation of prostate cancer cell [24, 25]. In addition, COL1A2 overexpression was a significant risk factor for intracranial aneurysm susceptibility [26]. Whereas, COL1A2 decreased the proliferative activity of liver epithelial cells [27]. These results suggested that COL1A2 was a double-edged sword in the biological process. This study evaluated the utility of COL1A2 as a potential biomarker in COAD and we explored the underlying mechanisms by which COL1A2 affected COAD through enrichment analysis.

Firstly, we had a general overview of COL1A2 from the following aspects: the differential mRNA expression of COL1A2 in various tumors, the location of COL1A2 protein in human tumor cells, and the different mutation types related to the COL1A2 gene. We found that COL1A2 was differentially expressed in most of the tumors, and the COL1A2 protein was located in the endoplasmic reticulum. The missense substitution of COL1A2 had the highest proportion of the mutation type. Then, we explored the COL1A2 expression in COAD and observed significant upregulation of COL1A2 transcriptional levels in COAD, and tended to increase along with stages and the nodal metastasis status. Epigenetic changes in genes are thought to be the leading cause of neoplastic transformation [28], and hence we analyzed the promoter methylation level of COL1A2 across various clinicopathological characteristics and found the possible negative correlation with the expression profile, particularly in terms of cancer stage and nodal metastasis status.

Next, we explored the relationship between COL1A2 mRNA expression and the clinical outcomes of COAD patients. Upon the survival analyses, COL1A2 overexpression contributed to a lower survival probability of DSS, OS, and PFS. Meanwhile, COL1A2 exhibited satisfactory performance in predicting the DSS, OS, and PFS status of patients with COAD. Further, Cox regression analyses showed that the elevated mRNA level of COL1A2 was an independent predictor for worse DSS and OS, revealing COL1A2 as a potential prognostic biomarker for COAD. Changes in COL1A2 in the ECM microenvironment are often accompanied by stromal invasion and the occurrence of epithelial neoplasms [29]. It is also involved in the induction of epithelial-mesenchymal transformation (EMT) in breast and lung cancer cells [30, 31]. Importantly, COAD arises from the epithelial cells of the colon or rectum, whose functions such as cellular differentiation, migration, and invasion are regulated by the physical interactions with ECM [29, 32]. Thus, it is reasonable to speculate that COL1A2 might promote tumorigenesis, invasion, and metastasis of COAD, which might be an explanation for the high expression of COL1A2 leading to a worse prognosis for COAD patients. Additionally, COL1A2 was secreted by stromal fibroblasts, while tumor-associated fibroblasts stimulate the occurrence of COAD in part by inducing inflammation in the tumor microenvironment [33–35]. The cancer cells and stromal cells in the COAD microenvironment generate high levels of pro-inflammatory eicosanoids, which are robust lipid mediators implicated in cancer cell angiogenesis, proliferation, and metastasis by the following mechanisms: direct activation of receptors on cancer cells; enforcing the epithelial cells to release angiogenic factors, pro-inflammatory mediators, and tumor growth factors [36]. This might be another explanation for the worse clinical outcomes of COAD patients with COL1A2 overexpression.

**Figure 10:**
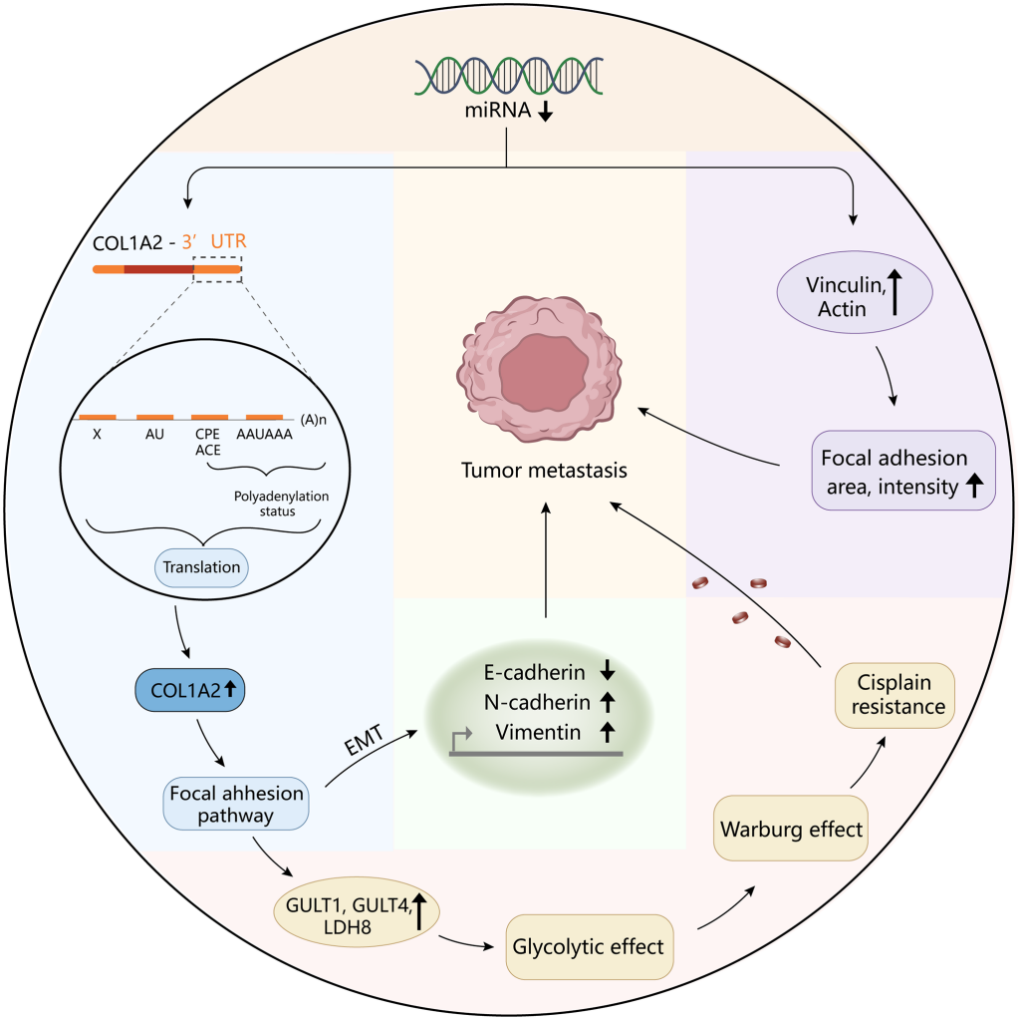
The diagram revealed the possible mechanism of COL1A2 involvement in colon adenocarcinoma.

To reveal the pathological function of COL1A2 in COAD, enrichment analyses were performed. GO enrichment of the COL1A-related DEGs showed the cellular response to an organic substance, extracellular matrix organization, extracellular matrix, vesicle, signaling receptor binding, and growth factor binding. The KEGG pathway showed the enrichment signaling pathways of oxidative phosphorylation, phagosome, focal adhesion, and fatty acid metabolism, among which focal adhesion shared the same pathway with GSEA results. Interestingly, COL1A2 expression was positively correlated with the enrichment levels of the focal adhesion pathway. Focal adhesion plays a vital role in cancer metastasis, invasion, and drug resistance [37]. Ning et al. reported that the focal adhesion signaling pathway was crucial in the EMT process in pancreatic cancer [38]. The activation of the focal adhesion pathway alters cancer cell glycolysis and induces cisplatin resistance in cervical and breast cancer [39]. Similarly, the authors inferred that COL1A2 might promote metastasis, and induce drug resistance, thereby enhancing the progression of COAD via regulation of the focal adhesion pathway. For further mechanistic explorations, Pekow et al. reported that COL1A2 was predicted to be the target of miR-4728-3p. In the HCT116 human colon cancer cells, miR-4728-3p regulated the COL1A2 gene expression by targeting the wild type 3′untranslated region (3′UTR) of the COL1A2 gene in the focal adhesion pathway. Besides, they revealed that miR-4728-3p decreased focal adhesion intensity and area [40]. Taken together, the possible mechanism of COL1A2 involvement in COAD was shown in Figure 10, which should be verified in the future. Additionally, other molecules could regulate COL1A2 mRNA expression. Acute ulcerative colitis (UC)-like colitis was induced in *Chga*-C57BL/ 6-deficient (*Chga^−/−^*) and wild-type (*Chga^+/+^*) mice. COL1A2 mRNA expression was decreased in *Chga^−/−^* mice model [41]. It has also been demonstrated that patients with UC are at high risk to develop colonic neoplasia [42]. Therefore, the authors speculated that targeting CHGA might decrease the COL1A2 mRNA expression, inhibiting the progression of COAD.

In summary, our study comprehensively enlightens the role of COL1A2 in the initiation and development of COAD. COL1A2 might serve as a valuable prognostic biomarker and a potential therapeutic target for COAD. Moreover, COL1A2 might promote the progression of COAD by positive regulation of the focal adhesion pathway.
